# Urinary Antiseptics

**Published:** 1914-04

**Authors:** 


URINARY ANTISEPTICS. J. W. Thompson Walker, *British Med. Journ.*, September 13, 1913. In an instructive paper the author reviews the value of various urinary antiseptics, with special reference to urotropin. The action of this drug depends upon its being split up into formaldehyde and ammonia. This reaction is accomplished in the urine itself when and only when that fluid is of acid reaction· An acidity of the urine which will only just turn litmus paper red will probably not cause any splitting of urotropin into its constituents; the urine must therefore be markedly acid. The normal acidity of the urine is due to acid sodium phosphate, and this body given as a drug is recommended as the best method of rendering the urine acid, though sometimes ammonium benzoate (15 gr.) may be found more efficacious.

Much controversy has raged as to this necessity of rendering the urine acid. The writer regards this as due to the lack of an adequate test for distinguishing formaldehyde from urotropin, which has resulted in an ignorance of the value of an acid urine. In the majority of his cases he checks the conversion of urotropin into formaldehyde by the following method, which he considers very satisfactory, as it will detect formaldehyde in 1 in 150.00. Three solutions are used: (1) Phenylhydrazine hydrochloride 0.5 per cent·; (2) sodium nitroprusside 5 per cent.; (3) sod. hydrate, saturated solution. Three drops of each of the first two solutions are added to the urine and a few drops of the sodium hydrate solution poured along the side of the test tube. A deep greenish-black colour indicates formaldehyde. This changes to green and fades through bright green and yellow. The fluids must be warm. If these tests are negative boil a fresh sample of urine c.$H_2So_4$ and repeat the test. If formaldehyde is now present the urine has contained urotropin.

The doses used are usually larger than those commonly employed, especially in cases of long standing, but he generally starts with fairly small ones and increases in some cases up to 25 gr. t·i.d. He considers, however, that it is better to give the drug in smaller doses and more frequently in cases where there is frequent micturition. In addition to insufficient acidity two causes render the administration of urotropin ineffective: (1) idiosyncrasy; (2) renal insufficiency.

Combinations of urotropin are of two types: (1) with an acid as helmitol (critic acid) or hetraline (resorcin); (2) with an alkali as cystopurin. The administration of the original drug with acid sodium phosphate will be found superior to either set of combinations.—J. B. Macalpine, in *Med. Chron.*, December, 1913.

Sphygometric Oscillometer of V. Pachon, applied to the diagnosis of arterial permeability. J .Guyot and G. Jeanneney, *Jour de Med. de Bordeaux,* September 21, 1913, allude to Moskowicz's suggestion in 1907 that the surface temperature be employed to diagnose the degree of arterial circulation but prefer the oscillometer. They cite a case of incipient gangrene of the left big toe in which the maxima and minima of oscillation of the pulse, showing the following results: Radial artery; 20-10; right dorsalis pedis 12-5; left dorsalis pedis 8-5; right tibio-peroneal trunk 15-4; left 10-5. The readings were the same on two consecutive days, on the second, the artery of the left instep showed an oscillation between 9 and 4.

